# Supplementary material for: Computational Modeling of Patient-Specific Healing and Deformation Outcomes Following Breast-Conserving Surgery Based on MRI Data
Source: Ann Biomed Eng. 2025 Nov 13;54(2):495–513. doi: 10.1007/s10439-025-03902-z (PMC12852230; doi:10.1007/s10439-025-03902-z)
Supplement: Supplementary file 1 — (pdf 4031 KB) [file 10439_2025_3902_MOESM1_ESM.pdf]

Supplementary Information for: Computational modeling of patient-specific healing and deformation outcomes following breast-conserving surgery based on MRI data

Zachary Harbin[1], Carla Fisher[2], Sherry Voytik-Harbin[3,4], Adrian Buganza Tepole[1,3*]

[1]School of Mechanical Engineering, Purdue University, West Lafayette, IN, USA.
[2]Division of Breast Surgery, Indiana University School of Medicine, Indianapolis, IN, USA.
[3]Weldon School of Biomedical Engineering, Purdue University, West Lafayette, IN, USA.
[4]Department of Basic Medical Sciences, Purdue University, West Lafayette, IN, USA.

*Corresponding author(s). E-mail(s): abuganza@purdue.edu;

Table 1 Parameters for the computational mechanobiological model.

Parameter	Description	Value	Reference
ρ_0 $[cells/mm^3]$	Nominal Fibroblast Density	55051	[1]
$d_{\rho,\phi}$ $[-]$	Fibroblast Diffusion Scaling Constant	1582.3	[1]
Δ $[-]$	Skewness of Fibroblast Speed $v_\rho(\phi)$	0	[1]
$d_{\rho,c}$ $[mm^2/hr]$	Cytokine-Increased Fibroblast Diffusivity	6.12×10^{-3}	[1]
$d_{\rho,0}$ $[mm^2/hr]$	Baseline Fibroblast Diffusivity	6.12×10^{-5}	[1, 2]
p_ρ $[1/hr]$	Fibroblast Proliferation	9×10^{-4}	[1]
$p_{\rho,c}$ $[1/hr]$	Cytokine-Increased Proliferation	0.015314	[1]
$K_{\rho,c}$ $[-]$	Proliferation Saturation due to Cytokine	1×10^{-5}	[3]
$p_{\rho,e}$ $[1/hr]$	Mechanoregulation of Fibroblast Proliferation	$p_\rho/2$	[4]
$K_{\rho,\rho}$ $[-]$	Fibroblast Division Saturation	$10 * \rho_0$	[5]
d_ρ $[1/hr]$	Fibroblast Death Rate	$p_\rho(1 - \rho_0/K_{\rho\rho})$	[5]
c_0 $[g/mm^3]$	Initial Cytokine Concentration Inside Cavity	1×10^{-4}	[3]
D_c $[mm^2/hr]$	Cytokine Diffusion Coefficient	0.01208	[6–8]
$p_{c,\rho}$ $[1/hr]$	Fibroblast Secretion of Cytokine	1.635×10^{-18}	[3]
$p_{c,e}$ $[1/hr]$	Mechanoregulation of Cytokine	5.45×10^{-18}	[3]
$K_{c,c}$ $[mol/mm^3]$	Cytokine Saturation	1	[3]
d_c $[1/hr]$	Cytokine Death Rate	0.005	[1]
k_0 $[MPa]$	Linear Stiffness	$[3.356 \times 10^{-3}$, $1.342 \times 10^{-2}]$	Dependent on Patient-Specific Breast Density
k_1 $[MPa]$	Compressibility	$[0.1667, 0.6667]$	Dependent on Patient-Specific Breast Density
k_f $[MPa]$	Fiber Stiffness	0.015	[9]
k_2 $[-]$	Nonlinear Stiffening	0.048	[9]
γ_e $[-]$	Shape of Mechanosensing Curve	5	[3]
ϑ_e $[-]$	Midpoint of Mechanosensing Curve	2	[3, 10]
t_ρ $[MPa]$	Contractile Force of Fibroblasts	2.33548×10^{-7}	[1]
$t_{\rho,c}$ $[MPa]$	Contractile Force of Myofibroblasts	$3.28571 \times t_\rho$	[1]
$K_{t,c}$ $[-]$	Traction Saturation due to Cytokine	1×10^{-5}	[3]
K_t $[-]$	Saturation of Mechanical Force by Collagen	0.2	[1]
p_ϕ $[1/hr]$	Collagen Production	1.4×10^{-8}	[1]
$p_{\phi,c}$ $[1/hr]$	Collagen Production Activated by Cytokine	7.0×10^{-8}	[1]
$K_{\phi,c}$ $[-]$	Collagen Production Saturation due to Cytokine	1×10^{-4}	[3]
p_{ϕ_e} $[1/hr]$	Collagen Production Activated by Stretch	p_ϕ	[3]
$K_{\phi,\rho}$ $[-]$	Collagen Production Saturation due to Collagen Fraction	1.06	[3]
d_ϕ $[1/hr]$	Collagen Degradation	$p_\phi(\rho_0/(K_{\phi,\rho} + 1))$	[10]
$d_{\phi,c}$ $[1/hr]$	Collagen Degradation Activated by Cytokine	8.81×10^{-5}	[11]
τ_{λ^p} $[1/hr]$	Rate of Plastic Deformation	0.05	[1]
τ_ω $[hr]$	Time Constant for Reorientation	$10/(K_{\phi,\rho} + 1)$	[3]
τ_κ $[hr]$	Time Constant for Dispersion	$1/(K_{\phi,\rho} + 1)$	[3]
γ_κ [-]	Shape of Dispersion Rate Curve	2	[3]

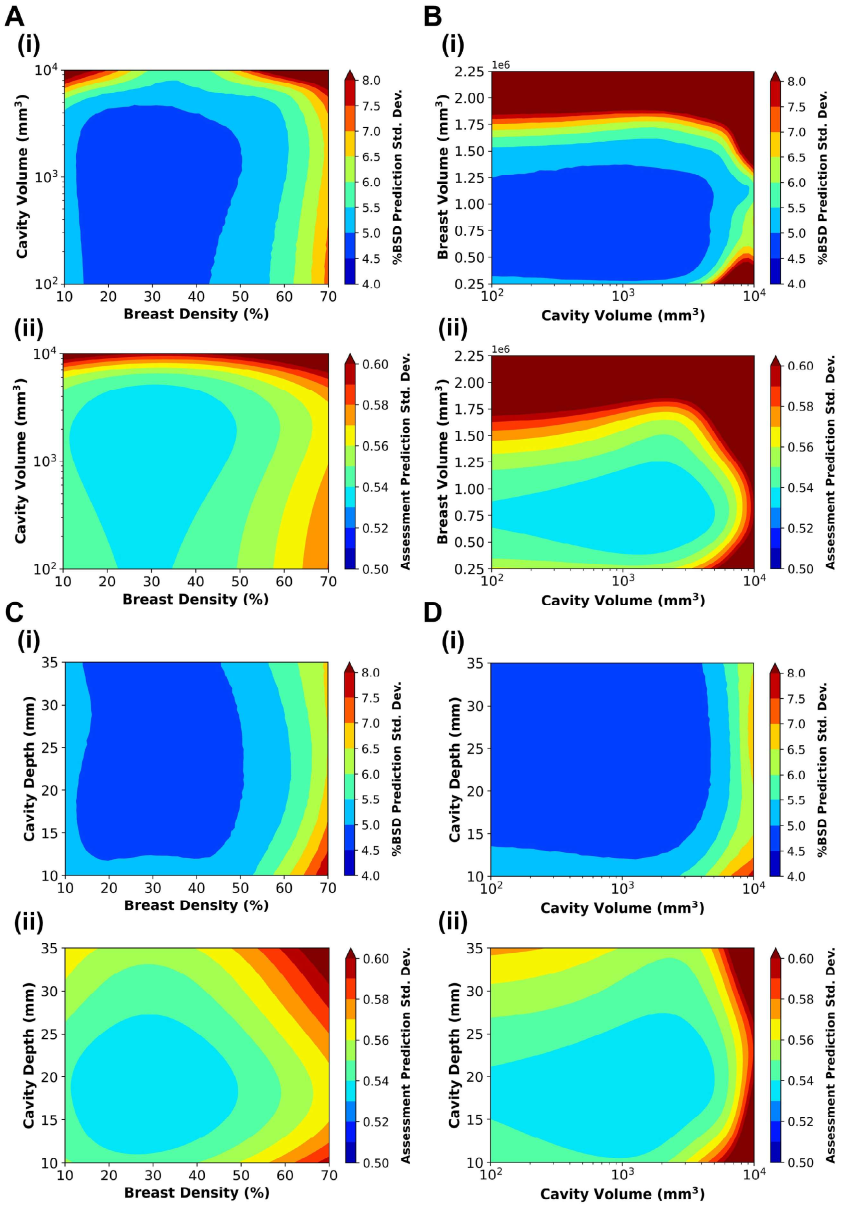

Fig. 1 Uncertainty in the Gaussian process surrogate model predictions corresponding to Fig. 7. Plots illustrate uncertainty, expressed as prediction standard deviations, in breast surface deformation predictions as a function of breast density, cavity volume, breast volume, and cavity depth, shown for two outcome metrics: **(i)** the percentage of the breast surface deforming more than 1 mm (%BSD), and **(ii)** the presence of visible post-surgical deformation (i.e., divot or indentation). Plots were generated using predictions from the multi-fidelity Gaussian process surrogate model and the Gaussian process classifier, respectively, by varying two of the four patient characteristics at a time. Median values for the remaining characteristics, derived from MRI data and summarized in Table 1, were held constant during the analysis.

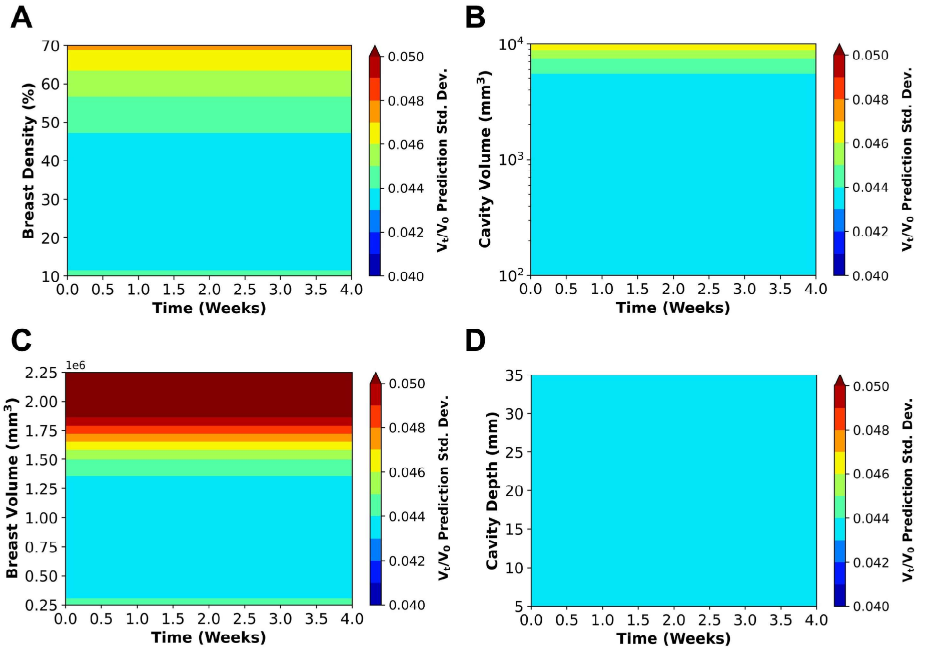

Fig. 2 Uncertainty in the Gaussian process surrogate model predictions corresponding to Fig. 8. Plots illustrate uncertainty, expressed as prediction standard deviations, in time-dependent cavity contraction predictions as a function of **A** breast density, **B** cavity volume, **C** breast volume, and **D** cavity depth. Plots were generated using the multi-fidelity GP surrogate model by varying one parameter at a time to isolate its influence. The remaining patient characteristics were held constant at their median values, derived from processed MRI data and summarized in Table 1.

References

[1] Harbin, Z., Sohutskay, D., Vanderlaan, E., Fontaine, M., Mendenhall, C., Fisher, C., Voytik-Harbin, S., Tepole, A.B.: Computational mechanobiology model evaluating healing of postoperative cavities following breast-conserving surgery. Computers in Biology and Medicine **165**, 107342 (2023)

[2] Olsen, L., Sherratt, J.A., Maini, P.K.: A mechanochemical model for adult dermal wound contraction and the permanence of the contracted tissue displacement profile. Journal of Theoretical Biology **177**(2), 113–128 (1995)

[3] Tepole, A.B.: Computational systems mechanobiology of wound healing. Computer Methods in Applied Mechanics and Engineering **314**, 46–70 (2017)

[4] Sohutskay, D.O., Tepole, A.B., Voytik-Harbin, S.L.: Mechanobiological wound model for improved design and evaluation of collagen dermal replacement scaffolds. Acta Biomaterialia **135**, 368–382 (2021)

[5] Valero, C., Javierre, E., García-Aznar, J.M., Gómez-Benito, M.J.: A cell-regulatory mechanism involving feedback between contraction and tissue formation guides wound healing progression. PloS One **9**(3), 92774 (2014)

[6] Cumming, B.D., McElwain, D., Upton, Z.: A mathematical model of wound healing and subsequent scarring. Journal of The Royal Society Interface **7**(42), 19–34 (2010)

[7] Koppenol, D.C., Vermolen, F.J., Niessen, F.B., Zuijlen, P.P., Vuik, K.: A mathematical model for the simulation of the formation and the subsequent regression of hypertrophic scar tissue after dermal wounding. Biomechanics and Modeling in Mechanobiology **16**, 15–32 (2017)

[8] Murphy, K.E., Hall, C.L., Maini, P.K., McCue, S.W., McElwain, D.S.: A fibrocontractive mechanochemical model of dermal wound closure incorporating realistic growth factor kinetics. Bulletin of Mathematical Biology **74**, 1143–1170 (2012)

[9] Tepole, A.B., Kabaria, H., Bletzinger, K.-U., Kuhl, E.: Isogeometric kirchhoff–love shell formulations for biological membranes. Computer Methods in Applied Mechanics and Engineering **293**, 328–347 (2015)

[10] Tepole, A.B., Ploch, C.J., Wong, J., Gosain, A.K., Kuhl, E.: Growing skin: a computational model for skin expansion in reconstructive surgery. Journal of the Mechanics and Physics of Solids **59**(10), 2177–2190 (2011)

[11] Laurent, G.: Dynamic state of collagen: pathways of collagen degradation in vivo and their possible role in regulation of collagen mass. American Journal of Physiology-Cell Physiology **252**(1), 1–9 (1987)
